# Raman and XPS studies of ammonia sensitive polypyrrole nanorods and nanoparticles

**DOI:** 10.1038/s41598-019-44900-1

**Published:** 2019-06-11

**Authors:** Milena Šetka, Raúl Calavia, Lukáš Vojkůvka, Eduard Llobet, Jana Drbohlavová, Stella Vallejos

**Affiliations:** 1 Central European Institute of Technology, Brno University of Technology, Purkyňova 123, 612 00 Brno, Czech Republic; 2 Minos-Emas, Universitat Rovira i Virgili, Av. Països Catalans 26, 43007 Tarragona, Spain; 3 Scientific-Tecnical Resources Service, Universitat Rovira i Virgili, Av. Països Catalans 26, 43007 Tarragona, Spain; 4 Department of Microelectronics, SIX Research Centre, Faculty of Electrical Engineering and Communication, Brno University of Technology, Technická 3058/10, 61600 Brno, Czech Republic; 5 Instituto de Microelectrónica de Barcelona (IMB-CNM, CSIC), Campus UAB, Carrer dels Til·lers, 08193, Cerdanyola del Vallès, Barcelona, Spain

**Keywords:** Sensors and biosensors, Nanoparticles

## Abstract

Polypyrrole (PPy) nanorods (NRs) and nanoparticles (NPs) are synthesized via electrochemical and chemical methods, respectively, and tested upon ammonia exposure using Raman and X-ray photoelectron spectroscopy (XPS). Characterization of both nanomaterials via Raman spectroscopy demonstrates the formation of PPy, displaying vibration bands consistent with the literature. Additionally, XPS reveals the presence of neutral PPy species as major components in PPy NRs and PPy NPs, and other species including polarons and bipolarons. Raman and XPS analysis after ammonia exposure show changes in the physical/chemical properties of PPy, confirming the potential of both samples for ammonia sensing. Results demonstrate that the electrochemically synthesized NRs involve both proton and electron transfer mechanisms during ammonia exposure, as opposed to the chemically synthesized NPs, which show a mechanism dominated by electron transfer. Thus, the different detection mechanisms in PPy NRs and PPy NPs appear to be connected to the particular morphological and chemical composition of each film. These results contribute to elucidate the mechanisms involved in ammonia detection and the influence of the synthesis routes and the physical/chemical characteristics of PPy.

## Introduction

Nanomaterials belong to one of the most active research areas of science and technology due to their size dependant properties, which are beneficial in different applications including medicine, environment, energy and various industries. Specifically, gas sensitive nanomaterials have proven possessing enhanced functionalities with better sensitivity and stability. These materials have also shown versatility for their surface modification, improving the selective detection of different vapours and gases^1^. Inorganic materials based on metals^2^ and/or semiconducting metal oxides^3^ are the most commonly used and studied gas sensitive materials. However, functional conductive polymers (CPs) including polyaniline (PANI)^4,5^, polydiacetylene (PDA)^6,7^, poly(3,4-ethylenedioxythiophene) (PEDOT)^8^ and polypyrrole (PPy)^9^ have shown potential for a next generation of flexible and wearable gas sensors^10^. The main advantage of CPs lies in their capability to operate at room temperature, as opposed to semiconducting metal oxides, which need high temperatures to operate (typically between 200 °C and 500 °C^11^).

In particular, PPy has been synthesized in the form of nanoparticles^12,13^, nanowires^14,15^, nanorods^16^, nanotubes^17^ and nanoribbons^18^ generally by electrochemical (electro-polymerization) or chemical methods, with both referring to the oxidative polymerization of the monomer pyrrole (Py). While electro-polymerized PPy is achieved using an appropriate electrolyte solution (Py and an oxidizing agent) under constant applied voltage or current in an electrochemical cell, chemically polymerized PPy is achieved via wet chemical synthesis by the oxidation of Py with an oxidizing agent such as FeCl_3_. The final properties of PPy in both synthesis methods are strongly dependent on factors such as the Py concentration, type of oxidizing substance and solvent^19–21^. Apart from those factors, the electro-polymerization of PPy also depends on the applied voltage and current density^21^. The electrochemical synthesis enables a wide choice of oxidizing agents, and localized control over material thickness and geometry, whereas the chemical synthesis provides the ability to generate nanostructures in a continuous, rather than batch mode. The latter implies higher throughputs and simpler instrumentation^22,23^.

**Figure 1:**
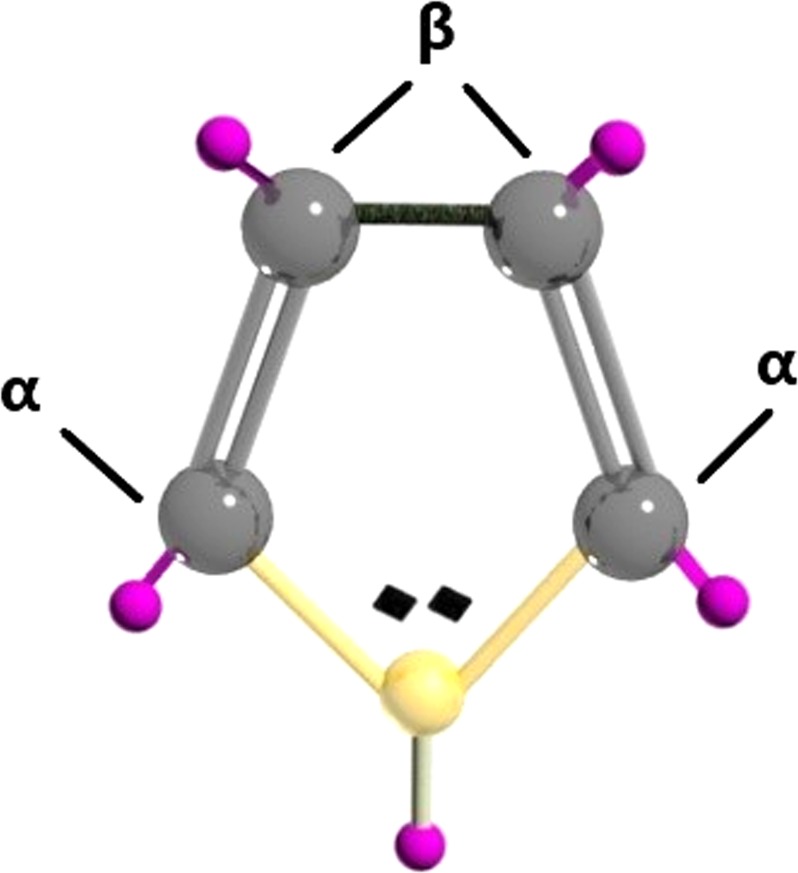
Molecular illustration of pyrrole monomer with the C atoms represented in grey, N atom in yellow and H atoms in purple colour.

Most of the chemical, electrical, and mechanical properties of PPy originate from its hetero-atomic and conjugated backbone structure (σ and π bonds)^11^, which contains polarons and/or bipolarons. These are oxidized states of PPy, associated with intermediate energy levels arose within the electronic band gap region of the polymer due to oxidation^24^). Polarons form upon oxidation, when a π-electron is removed from the neutral PPy chain, whereas bipolarons appear upon further oxidation, when a second electron is removed from the PPy chain^25^. This electron transfer occurring between PPy and the dopant anion (i.e., anion from the oxidizing agent) causes electron relocalization and in turn the structural lattice distortion of the PPy ring^26^. Additionally, the chemical, electrical, and mechanical properties of PPy can also be linked to its morphology (i.e., the physical organisation of the macromolecules on a microscopic scale), which is determined by the conformation and arrangement of the Py chain segments in space **(**Fig. 1**)**. The resulting PPy configuration is dependent on the synthetic route and variables such as the type of the dopant anions employed. Usually, Py rings tend to connect through α-positions leading to a planar configuration of PPy. However, Py rings can also connect through β-positions, or both α- and β-positions in the same ring leading to a nonplanar configuration, due to the steric crowding of the substituent hydrogen atoms, and the presence of defects in the structure^27,28^. Consequently, the sensing properties of PPy are influenced in part by its morphology as well as its oxidation level, which is readily affected by chemical or electrochemical doping/de-doping (oxidation/reduction) mechanisms during the exposure of PPy to the gas analyte. Spectroscopy techniques such as Raman and X-ray photoelectron spectroscopy (XPS) allow for the characterization of these chemical or electrochemical properties. Raman facilitates the analysis of the intermolecular interactions and XPS provides a semi-quantitative analysis of surface chemical composition.

PPy with different morphologies has been reported previously as being sensitive to diverse gases and vapours, particularly to ammonia^11^. Generally, the literature attributes these sensing properties to the transfer of protons or electrons between PPy and the gaseous analytes. However, often the correlation of these mechanisms with the morphology and synthesis method of PPy have been explored less^29,30^. Hence, here we get an insight using spectroscopy techniques such as Raman and XPS into the dominant gas detection mechanisms of PPy NRs and PPy NPs synthesized electrochemically and chemically, respectively, towards ammonia as a model gas molecule.

## Results and Discussion

### Physical and chemical characteristics of the PPy NRs and PPy NPs

**Figure 2:**
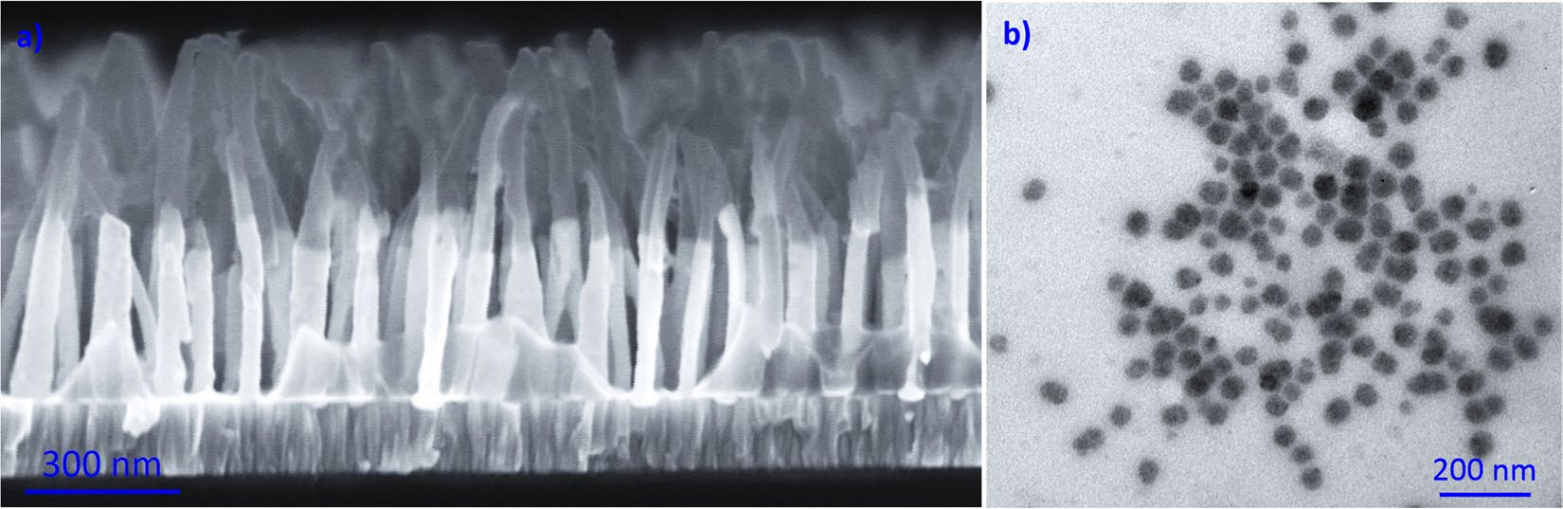
SEM and TEM images of the PPy NRs (**a**) and PPy NPs (**b**), respectively.

Figure 2 displays the morphology of the PPy NRs and PPy NPs observed via SEM and TEM, respectively. SEM of the PPy NRs (Fig. 2a) revealed quasi-aligned structures with a diameter of ~50 nm and a length of ~340 nm (grown on the top of 340 nm long Au NRs employed as template). TEM of the PPy NPs (Fig. 2b) proved the formation of homogeneously distributed compact spherical NPs with different sizes between 30 and 50 nm.

**Figure 3:**
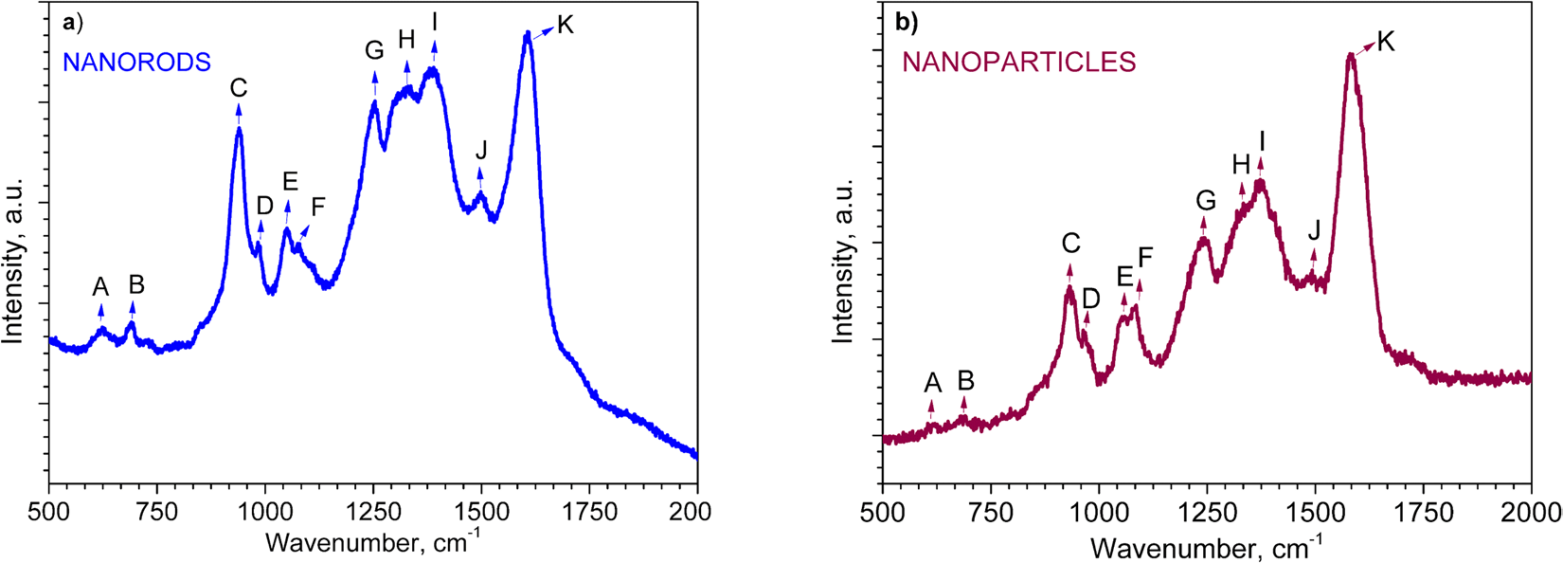
Raman spectra of the PPy NRs (**a**) and PPy NPs (**b**). The assignment of each peak is presented in Table 1.

Raman analysis of PPy NRs and PPy NPs at room temperature (Fig. 3) displayed various Raman bands between 620 and 1609 cm^−1^, consistent with those reported previously in the literature^31–42^. Table 1 shows a summary of the Raman peaks observed in Fig. 3 with the assigned predominant band vibrations. Both samples displayed similar Raman bands with slight shifts of less than 20 cm^−1^, most likely caused by the different laser wavelengths used for excitation of each sample (see experimental section).

**Table 1:** Assignment of Raman peaks for PPy NRs and PPy NPs in Fig. 3.

Band	Wavenumbers, cm^−1^	Assignment
A	620–626	C–C ring torsional
B	687–690	C–H wagging
C	933–939	C–C ring deformation (bipolarons)
D	963–985	C–C ring deformation (polarons)
E	1050–1056	C–H in-plane deformation (polarons)
F	1079–1084	C–H in-plane deformation (bipolarons)
G	1241–1253	Antisymmetric C–H in-plane bending, ring stretching
H	1330–1333	C–C in-ring, antisymmetric C–N stretching
I	1372–1389	C–C in-ring, antisymmetric C–N stretching, C–H bending, N–H bending stretching
J	1492–1498	C–C, C=N stretching
K	1583–1609	C=C in-ring, C–C inter-ring stretching

**Figure 4:**
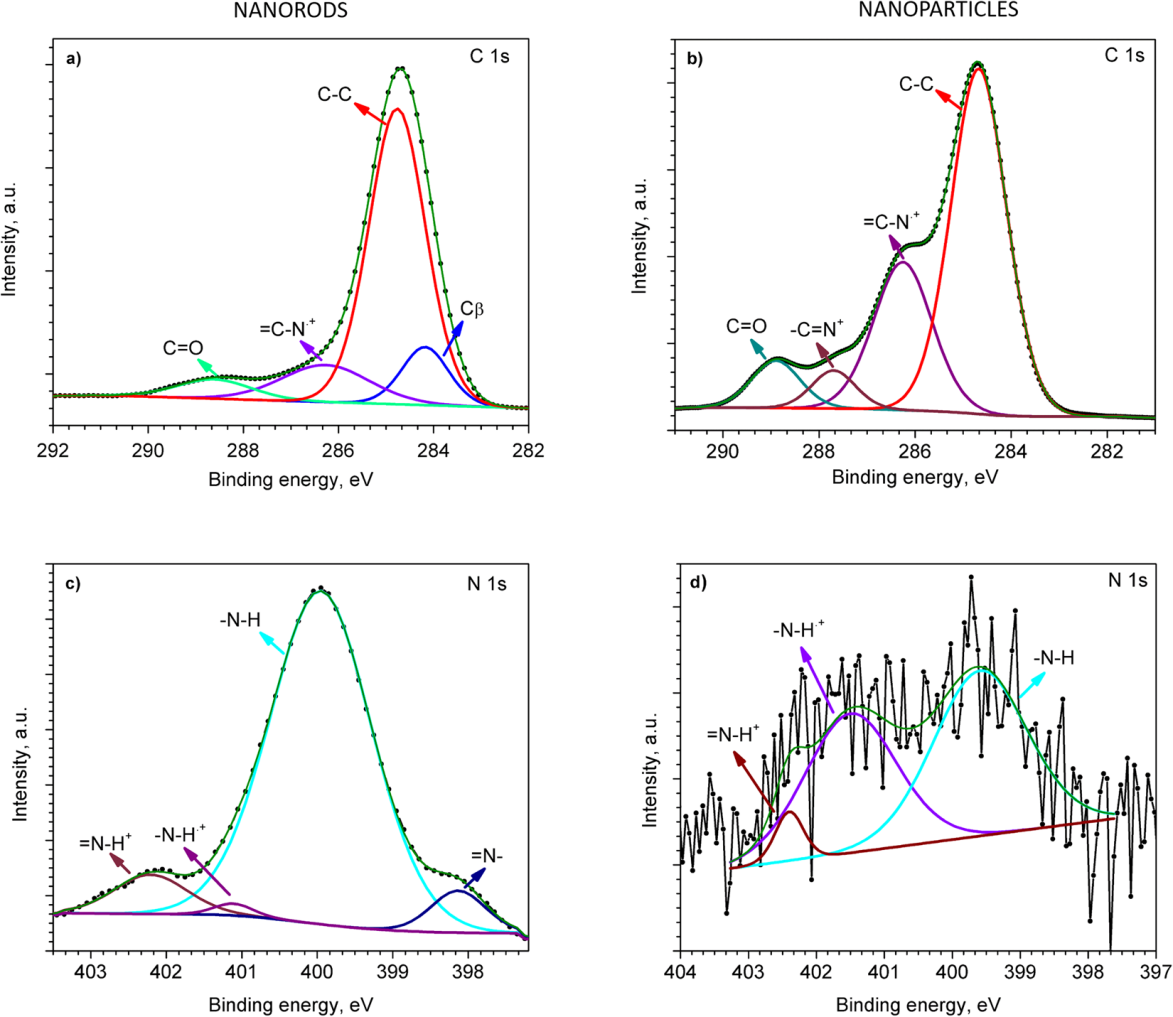
C 1s (**a**,**b**) and N 1s (**c**,**d**) XPS core level peaks recorded on the PPy NRs (left) and PPy NPs (right).

XPS analysis of the films showed characteristic C 1s, and N 1s core level peaks both for the PPy NRs and PPy NPs (Fig. 4). Further examination of the C 1s core level peaks and their deconvolution into Gaussian components revealed different characteristics for the PPy NRs and PPy NPs. Thus, the C 1s spectrum for the PPy NRs films consist of four components (Fig. 4a) with the most intense peak at 284.7 eV. This peak corresponds to α carbon atoms in the Py ring (carbon atom bonded to a functional group, Fig. 1), which are presented as C–C, C–H or C=C^43,44^. In contrast, the component at 284.2 eV is linked to β carbon atoms (carbon atoms that are not bonded to the functional group, Fig. 1)^9^. The component centred at 286.3 eV is related to the bonds between carbon and nitrogen in PPy structures, specifically attributed to the = C–N^.+^ bond of PPy polarons^45^. Finally, the fourth component with the smallest area, appearing at the highest binding energy (288.6 eV), is assigned to C=O species^46^. Similarly, the C 1s core level peak recorded on the PPy NPs showed four components (Fig. 4b). Three of these components correspond to α carbon atoms, the = C–N^.+^ bond of PPy polarons, and the C=O species identified in the PPy NRs, whereas the fourth component at 287.7 eV corresponds to the –C=N^+^ bond of bipolaron charge carrier species^45^.

The deconvolution of N 1s XPS core level peaks recorded on the PPy NRs and PPy NPs displayed different characteristics. For instance, the N 1s peak for PPy NRs showed four components (Fig. 4c), while the weak intensity N 1s peak recorded on the PPy NPs showed the presence of only three components (Fig. 4d). In line with the literature, the components at ~400 eV are assigned to neutral nitrogen in the Py ring (–N–H structures), whereas the components shifted to the higher binding energies are most likely related to the positively charged nitrogen of polaron (–N–H^.+^) and bipolaron (=N–H^+^) species. The component with the lowest binding energy, at 398 eV, corresponds to imine (=N–) structure^43,44,47,48^.

Both samples (PPy NRs and PPy NPs) indicated a major presence of α carbon atoms in the PPy ring matching with the planar conformation of PPy (i.e., Py rings linked by α-positions). However, the presence of β carbon atoms and deprotonated nitrogen atoms (=N–) in the PPy NRs (Fig. 4a,c) suggest a degree of disorder in this sample. This signifies a nonplanar PPy configuration deviated from the ideal arrangement of PPy, in which Py rings are linked only via the α-positions. This could be attributed in part to the use of a higher number of reactants in the electrochemical synthesis as compared to the chemical synthesis, which in turn results in the incorporation of a higher number of impurities^49,50^. Also, it could be attributed to the use of ‘spherical’ dopant anions such as BF_4_^−^ (tetraethylammonium tetrafluoroborate) for PPy NRs, which cause particular defects in PPy, leading to non-planar configuration and different orientation at the surface^51^. The XPS survey spectra for both types of PPy films and the estimation of the concentration of these elements at the surface is shown in Fig. S1 (found for PPy NRs: Au, B, P, Cr and F, found for PPy NPs: Cl, Si and Fe).

The O 1s core level spectrum was also recorded for both samples. O 1s core levels are present due to the PPy degradation by OH^•^ radicals. This oxygen is in the form of C–O–C and C=O moieties in the NRs and C=O and C–O in the NPs. The NPs also indicate the presence of iron oxide traces^46,52,53^ (Fig. S2).

### Gas sensing properties

To get an insight into the sensing properties of the PPy NRs and PPy NPs, sensing tests with *in-situ* Raman spectroscopy were performed towards various gas analytes, including ammonia, ethanol, acetone, and toluene. Generally, results revealed repeatable and marked changes in the PPy bands of both samples (i.e. PPy NRs and PPy NPs) after ammonia exposure, contrary to the tests upon ethanol, acetone, and toluene, which showed negligible changes in the characteristic Raman bands of both samples. Thus, further gas sensing tests were focused on ammonia (0.3%) as a model analyte. Other ammonia concentrations were also analysed without noticing significant changes in PPy bands.

Previous reports in the literature suggest that the detection mechanism of PPy towards ammonia occurs either via proton transfer (deprotonation) and/or electron transfer (electron injection)^54^. In the proton transfer reaction, the protonated form of PPy (–N^+^–H– site) loses protons, whereas the nitrogen atoms of NH_3_ establish coordination bonds with free atomic orbital of dopant X^−^ (e.g., BF_4_^−^ for the NRs and Cl^−^ for the NPs), which leads to the deprotonation of PPy and creation of ammonium salt. This reaction can be described as follows^54^:

1 $${{\rm{PPy}}}^{+}{{\rm{X}}}^{-}+{{\rm{NH}}}_{3}\rightleftharpoons {\rm{PPy}}{(-{\rm{H}})}^{0}+{{{\rm{NH}}}_{4}}^{+}{{\rm{X}}}^{-}$$

Alternatively, the electron transfer can occur via reversible transformation of the positively charged PPy backbone (PPy^+^) into the neutral form (PPy^0^). Thus, when PPy is exposed to ammonia, a lone pair of electrons on the nitrogen atom of ammonia causes the reduction of PPy backbone (see Supplementary Information, Fig. S3)^38^. This electron transfer reaction could be written as follows^54^:

2 $${{\rm{PPy}}}^{+}{{\rm{X}}}^{-}+{{\rm{NH}}}_{3}\rightleftharpoons {{\rm{PPy}}}^{0}/{{{\rm{NH}}}_{3}}^{+}{{\rm{X}}}^{-}$$

**Figure 5:**
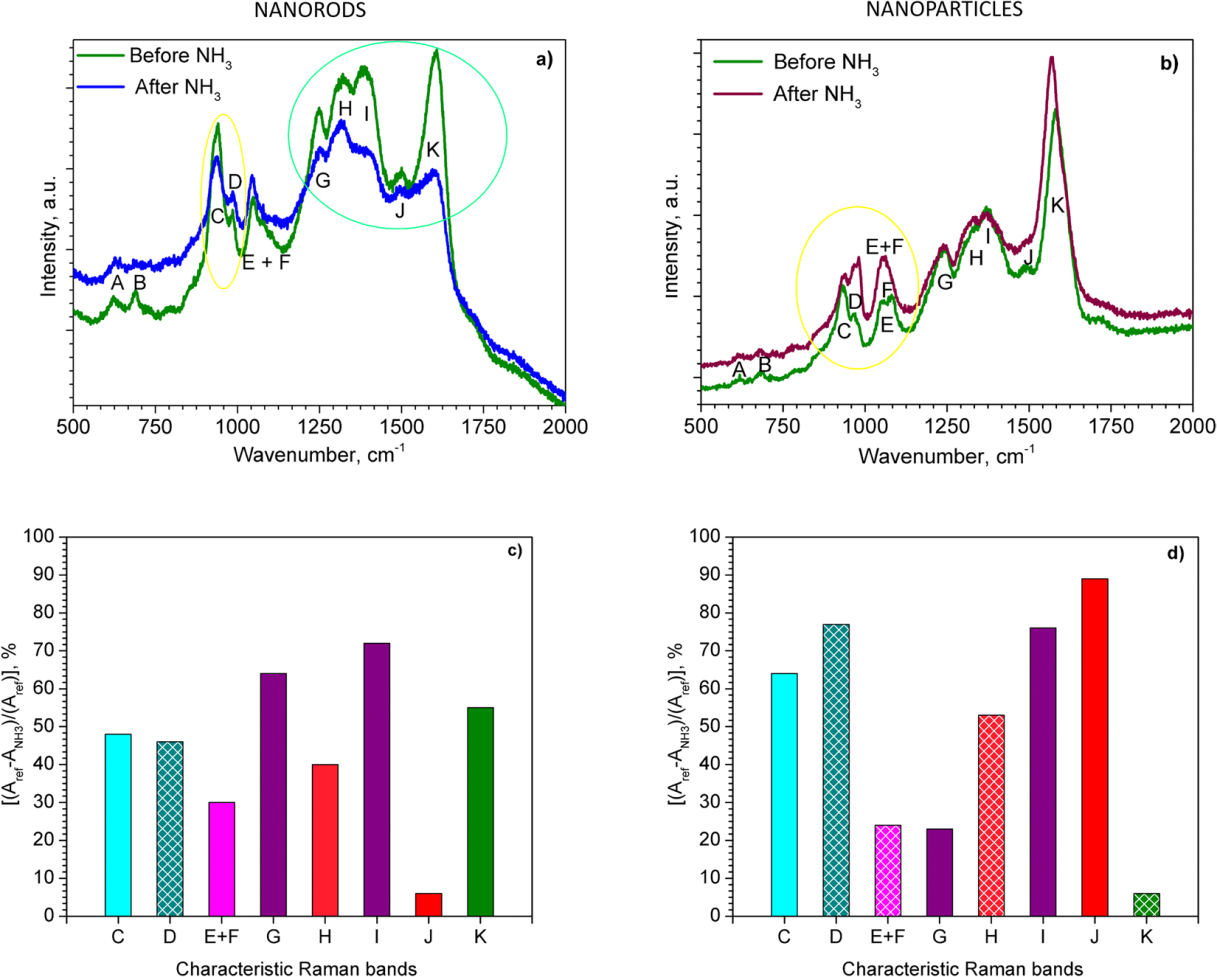
Raman spectra of the PPy NRs (**a**) and PPy NPs (**b**) before (green line) and after the exposure to ammonia (blue and brown line); Relative changes of each Raman band area [(*A*_ref_ − *A*_NH3_)/(*A*_ref_)] for the PPy NRs (**c**) and PPy NPs (**d**) after ammonia exposure. The area of the corresponding band (i.e., C, D, E, *etc*.) before and after ammonia exposure is expressed by *A*_ref_ and *A*_NH3_, respectively. The patterned columns represent an increase in peak area after ammonia exposure, whereas the full color columns represent a decrease.

Figure 5a,b compare the Raman bands recorded before (reference spectra) and after exposing the PPy NRs and PPy NPs to ammonia (the characteristic Raman bands are labelled according to Table 1). Overall, the spectra after ammonia exposure revealed a slight shift (less than 10 cm^−1^) to lower wavenumbers. This shift, however, was fully recovered after removing ammonia from the sample enviroment, which indicated a good reversibility of the system. The Raman bands (highlighted in green and yellow circles) in Fig. 5a,b show significant changes in the intensity and the full width at half maximum (FWHM) with respect to the reference.

The column chart for the PPy NRs in Fig. 5c compares the relative change of area for each Raman band after ammonia exposure. The changes are particularly marked for the C, G, H, I and K vibration bands which display approximately 48, 64, 40, 72 and 55% decrease in peak area after ammonia exposure, respectively. On the contrary, the D band is the only vibration band showing an increase in peak area and indicates a change of approximately 46% after ammonia exposure. The diminution and broadening of the I band, which is modulated in part by the intensity of various modes (see Table 1) has been demonstrated previously to be indicative of the deprotonation of PPy^38^. Consequently, this shows the presence of a mechanism in which protons from –N^+^–H– site of PPy NRs are transferred to ammonia^55^. In contrast, the changes observed in the bands C and D which correspond to polaron and bipolaron species, suggest the presence of an electron transfer mechanism in PPy NRs during ammonia exposure. This is consistent with the decrease of C peak (bipolaron) and increase of D peak (polaron) which indicates the conversion of bipolarons into polarons via the donation of electrons from ammonia. The presence of both mechanisms (i.e. proton and electron transfer) causes a new resonance structure in the ring^38^ and in turn, the changes in the G, H, I and K bands as noticed in Fig. 5a,c. Thus, the new rearranged PPy structure may be characterized by different angles among the atoms and length of the bonds compared to the original PPy structure (i.e., before ammonia exposure).

**Figure 6:**
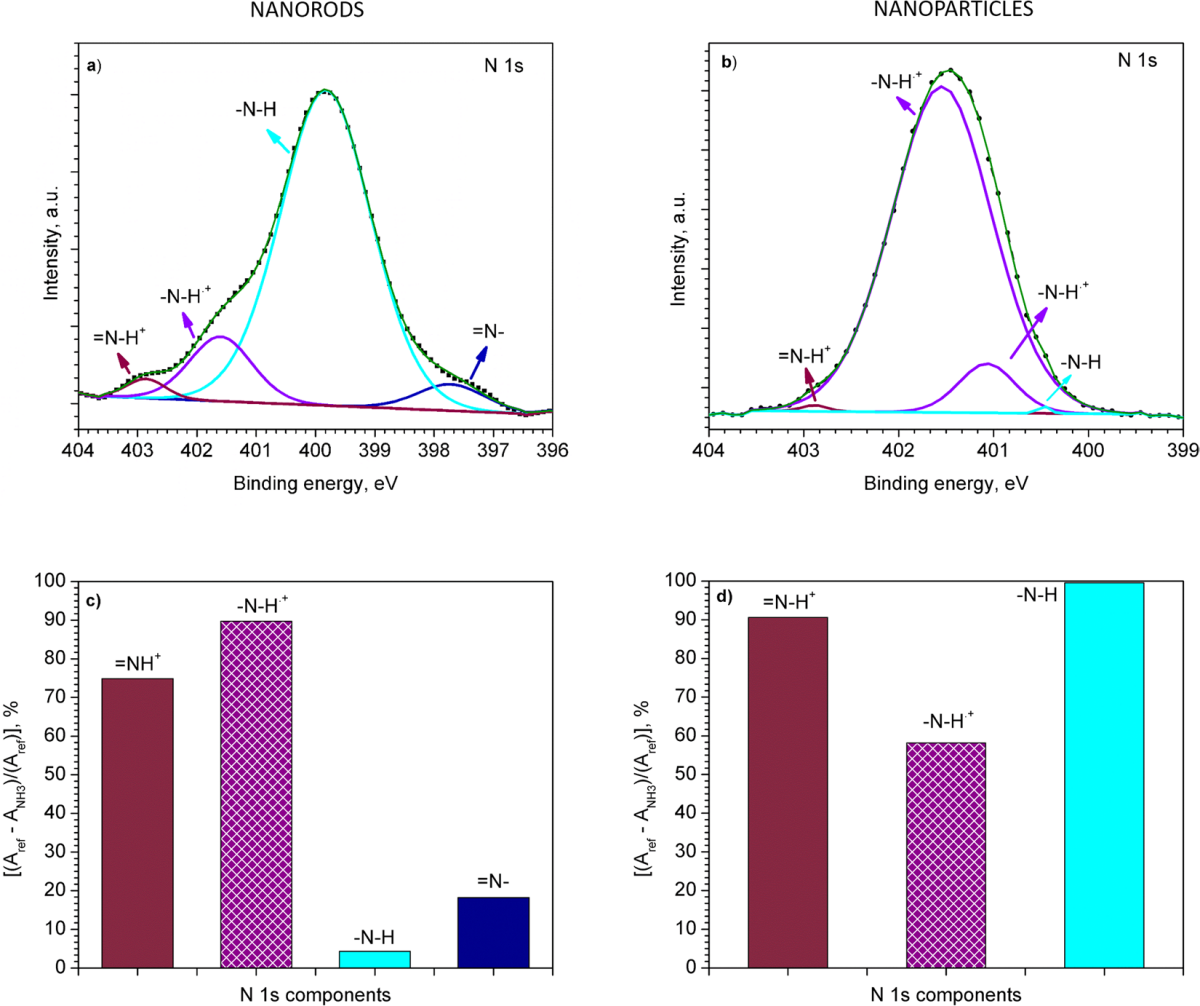
N 1s core level XPS spectra for the NRs (**a**) and NPs (**b**) after the exposure to ammonia. Relative change in area [(*A*_ref_ − *A*_NH3_)/(*A*_ref_)] of each PPy component after ammonia exposure (NRs (**c**) and NPs (**d**)). The area of the corresponding components (i.e., –N–H, –N–H^.+^, *etc*.) before and after ammonia exposure is expressed by *A*_ref_ and *A*_NH3_, respectively. Patterned and full color columns represent an increase or decrease in peak area after ammonia exposure, respectively.

**Figure 7:**
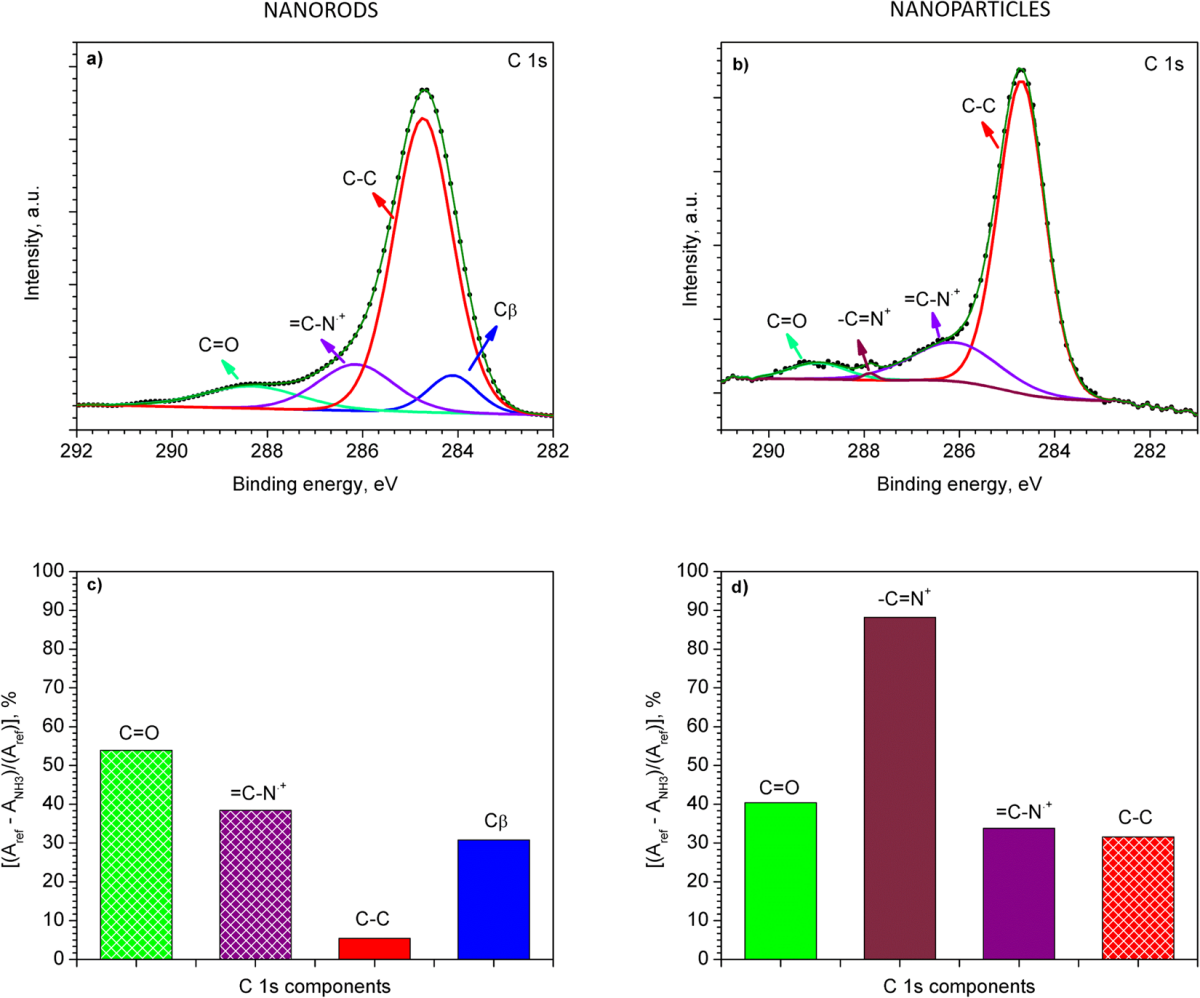
C 1s core level XPS spectra for the NRs (**a**) and NPs (**b**) after the exposure to ammonia. Relative change in area [(*A*_ref_ − *A*_NH3_)/(*A*_ref_)] of each PPy component after ammonia exposure (NRs (**c**) and NPs (**d**)). The area of the corresponding components (i.e., C_β_, C–C, *etc*.) before and after ammonia exposure is expressed by *A*_ref_ and *A*_NH3_, respectively. Patterned and full color columns represent an increase or decrease in peak area after ammonia exposure, respectively.

Further XPS analysis of the PPy NRs after ammonia exposure also confirmed the presence of an electron transfer mechanism. After ammonia exposure, the XPS results revealed an increase of polarons and simultaneous decrease of bipolarons species in the N 1s core level peak **(**Fig. 6a). For instance, after ammonia exposure, the ratio of the components related to polarons and bipolarons was 6 times higher than the ratio recorded before ammonia exposure (Fig. 4c). The relative changes in area of the characteristic component after ammonia exposure are displayed in Fig. 6c. Similarly, the C 1s core level XPS spectrum (Fig. 7a) after ammonia exposure also indicates an increase of =C–N^.+^ (polaron) species (Fig. 7c), with a slight change in the relation of β carbon atoms, C–C and C=O components (Fig. 7c) as compared to the components obtained before ammonia exposure (Fig. 4a). The presence of a proton transfer mechanism, as noticed via Raman for the PPy NRs after ammonia exposure, was not evidenced in the XPS analysis, probably due to the different principles of XPS and Raman. XPS allows observing the chemical and/or electronic states of the elements at the material surface (up to 10 nm in depth) and Raman analyzes vibrational, rotational, and other low-frequency modes within the bulk of the material.

As far as the PPy NPs results concern, the changes on the Raman spectra of PPy NPs after ammonia exposure are also manifested by relative changes of area in each band (Fig. 5b). The most noticeable changes after ammonia exposure are observed for the bands C, I and J with a decrease in peak area of 64, 76 and 89%, respectively. In contrast, the D and H bands showed an increase in peak area of 77 and 53%, respectively (Fig. 5d). The changes in the C and D Raman bands for PPy NPs after ammonia exposure indicate a decrease in the amount of bipolarons (C band) with simultaneous increase of polarons (D band). This fact is also supported by the disappearance of the bipolarons band (F) and the sharpness of the polarons band (E) after ammonia exposure. Previous works attributed the loss of the F band after reduction of oxidized PPy^41^. This statement is also consistent with the shift of the K band to the lower wavenumbers which also indicates a reduced (less oxidized) PPy structure^35^. Consequently, the conversion of bipolarons into polarons leads to a distortion of the bonds and angles along the PPy chain (Fig. S3). This is in agreement with the new resonance recorded for the H, I, and J bands after ammonia exposure (Fig. 5b). This new resonance is manifested by the loss of J band and the large changes in the peak area of H and I bands (Fig. 5d) which indicate the break of C=N double bond of bipolarons and the conversion to C–N single bonds of polarons.

The N 1s core level spectrum also demonstrates the presence of positively charged nitrogen (–N–H^.+^) of polaron species (Fig. 6b), with a strong decrease in the bipolaron (=N–H^+^) species and neutral nitrogen (–N–H) (Fig. 6d) after ammonia exposure. These results indicate a nine-fold increase of the polarons to bipolarons ratio as compared to the ratio registered before ammonia exposure. The C 1s XPS core level spectra of the PPy NPs after ammonia exposure (Fig. 7b) also points out to the conversion of bipolarons into polarons. XPS spectra (Figs 4b and 7b) show a lower polaron to bipolaron before (i.e., 7) than after (i.e, 35) ammonia exposure, indicating a five-fold increase in this ratio. These results are consistent with the Raman analysis described above, indicating that the PPy NPs exposed to ammonia follow a detection mechanism dominated by electron transfer, with no evidence of a proton transfer mechanism as noticed for the PPy NRs.

In summary, these results indicate that the ammonia detection mechanisms in PPy are prone to be adjusted by the synthesis route, namely by tuning reactants, concentrations, and/or applied charge density in the case of electrodeposition. These synthesis conditions at the first stage define the morphology, surface area and orientation of PPy. However, in a more advanced stage this influences the detection mechanisms of ammonia. Considering the specific synthesis conditions and gas analyte studied in this work, Raman and XPS tests evidenced electron transfer mechanisms in both, the electrochemically synthesized PPy NRs and the chemically synthesized PPy NPs, whereas the presence of detection mechanism in which protons are transferred was only evidenced in the case of PPy NRs. The possibility to tailor these mechanisms as a function of the synthesis route would be beneficial for specific sensing principles. For instance, a sensing based on mass-sensitive principle, in which the presence of proton-based mechanism could be more favourable considering that the relative mass of protons is higher (~2000 times more) than that of electrons.

## Conclusions

An electrochemical and chemical synthesis of polypyrrole to form rods (PPy NRs) and particles (PPy NPs) at nanoscale was developed. SEM and TEM analysis revealed PPy NRs with diameters of 50 nm and lengths of 300 nm, approximately, and PPy NPs with diameters between 30 and 50 nm. The mechanisms involved during the exposure to ammonia of these materials were addressed *in-situ*, using Raman and XPS analysis. Results demonstrated a detection mechanism dominated by both electron and proton transfer in the case of PPy NRs, as opposed to PPy NPs which showed a mechanism based on electron transfer. The results are affected in part by different morphological/structural properties of the films and by the incorporation of different impurities derived from the particular steps of each synthesis process. This proves a significant impact of the entire technological process on the detection behavior of the same material.

## Materials and Methods

### Electrochemical synthesis

PPy NRs were synthesized on the top of preformed gold nanorods (Au NRs)^56^ via template-based electropolymerization enabled by a two-electrode system under potentiostatic (constant potential) mode. Silicon wafers with thermally oxidized silicon dioxide layer (1.5 × 1.5 mm) covered, from bottom to top, by sputter-deposited titanium (Ti, 20 nm thick), tungsten (W, 150 nm thick) and aluminium (Al, 500 nm thick) were used as a substrate. The deposition of above mentioned layers was performed using RFICP Kaufman ion-beam source, KRI®.

In the first step, the electrochemical anodization of Al and W was performed under 40 V in 0.3 M oxalic acid at 10 °C. These conditions led to the formation of nanoporous anodized alumina oxide (AAO) template with tungsten trioxide (WO_3_) nanodots at the bottom which were subsequently etched in a phosphate buffer solution (pH = 7, T = 25 °C). This step allowed the formation of W nanodimpled surface as a base for the growth of the Au NRs. The pulsed galvanic deposition of gold (Au) was carried out in potassium dicyanoaurate solution under following conditions: 35 pulses, pulse length of 400 ms, a period of 2 s between pulses, a current of 1 mA, and a potential of 5 V.

Then PPy was deposited under a voltage of 2 V for 120 s during the electropolymerization. The PPy was formed in a mixture of Py monomer and anionic doping salt, tetraethylammonium tetrafluoroborate, with the molar ratio of 2:1, dissolved in acetonitrile solvent. To clean the surface and eliminate any over deposition of the PPy, the polymer layer was etched using oxygen plasma with applied power of 100 W at 50 mTorr and a gas flow of 50 sccm of O_2_ for 15 min. Finally, the AAO template was selectively dissolved in the aqueous solution of chromium trioxide and phosphoric acid at 60 °C for 600 s.

### Chemical synthesis

PPy NPs were obtained via oxidative chemical polymerization of Py monomer based on the formation of complex between water-soluble polymer poly vinyl alcohol (PVA) and iron cations (III) of FeCl_3_ in aqueous solution, as described previously^13^. Briefly, 7.5 wt% PVA was diluted in distilled water and stirred till all PVA was completely dissolved. Subsequently, 3.73 g of FeCl_3_ was added to the solution; after this step, a change of color from transparent to yellow was observed. The stirring was continued for 5 min until an equilibrium was established, then 0.69 cm^−3^ of Py monomer was dropped in the reaction mixture. As soon as the Py mixed with the oxidant (FeCl_3_), a rapid polymerization reaction occurred. This turned the solution into a characteristic black color that indicates the formation of PPy. The stirring process was kept constant for 5 h, subsequently the PPy NPs were separated from the solution via centrifugation (6000 RPM for 60 min) and diluted in ethanol. Finally, PPy NPs were drop coated on silicon tiles (1.5 × 1.5 mm) and dried at 80 °C for 30 min.

### Material analysis and gas tests

Raman analysis was carried out using a Renishaw InVia Raman microscope employing 785 nm and 633 nm laser beam for PPy NRs and PPy NPs, respectively. The spectra were recorded using a power lower than 1 mW (i.e., power density was 0.1627 mW/μm^2^ for PPy NRs and 0.0085 mW/μm^2^ for PPy NPs), and a 20× objective. Raman spectra were recorded at 60 °C. During the measurements, the samples were placed in a chamber equipped with a continuous gas flow system. Nitrogen was used as reference and simultaneously as a carrier gas for the saturated ammonia vapour which was produced by bubbling ammonia (Panreac, 30% w/v) at room temperature. The resulting ammonia concentration was contrasted using a commercial photoionization detector (MiniRAE30000 with an operating range of 0–1.5%). Thus, PPy NRs and PPy NPs samples were exposed either to nitrogen or 0.3% of ammonia for 5 min. Afterwards, the Raman spectra were recorded. After ammonia exposure, the samples were cleaned in N_2_ flow for 15 min. The Raman spectra before and after ammonia exposure presented in this work were recorded on the same sample and site. Moreover, the same procedure was repeated in various sites of the sample to confirm the tendency of the spectra.

In order to further study the mechanisms of ammonia detection, the PPy NRs and PPy NPs were exposed to ammonia flow for 20 min at 60 °C and subsequently, the XPS spectra were recorded. These analyses were performed on the same samples used for Raman. The XPS spectra before and after ammonia were measured in various nearby sites. XPS was carried out using Kratos AXIS Supra spectrometer with monochromatic Kα X-ray radiation, emission current of 15 mA, hybrid lens mode and charge compensation on. High-resolution spectra were collected with analyser pass energy of 20 eV and with 0.1 eV energy resolution. The deconvolution of all XPS data was elaborated using Casa XPS v.2.3.18 software (the spectra were calibrated with respect to the C 1s peak at 284.7 eV).

## Supplementary information

Supplementary info
