# Supplementary material for: Raman and XPS studies of ammonia sensitive polypyrrole nanorods and nanoparticles
Source: Sci Rep. 2019 Jun 11;9:8465. doi: 10.1038/s41598-019-44900-1 (PMC6559985; doi:10.1038/s41598-019-44900-1)
Supplement: Supplementary file 1 — Supplementary info [file 41598_2019_44900_MOESM1_ESM.pdf]

# Supplementary information

# Raman and XPS studies of ammonia sensitive polypyrrole nanorods and nanoparticles


Milena Šetka[1], Raúl Calavia[2], Lukáš Vojkůvka[3], Eduard Llobet[2], Jana Drbohlavová[1,4], Stella Vallejos[1,5*]

*[1]Central European Institute of Technology, Brno University of Technology, Purkyňova 123, 612 00 Brno, Czech Republic*
*[2]Minos-Emas, Universitat Rovira i Virgili, Av. Països Catalans 26, 43007 Tarragona, Spain*
*[3]Scientific-Tecnical Resources Service, Universitat Rovira i Virgili, Av. Països Catalans 26, 43007 Tarragona, Spain*
*[4]Department of Microelectronics, SIX Research Centre, Faculty of Electrical Engineering and Communication, Brno University of Technology, Technická 3058/10, 61600 Brno, Czech Republic*
*[5]Instituto de Microelectrónica de Barcelona (IMB-CNM, CSIC), Campus UAB, Carrer dels Til·lers, 08193, Cerdanyola del Vallès (Barcelona), Spain*
*Correspondence and requests for materials should be addressed to S.V. (email: vargas@feec.vutbr or stella.vallejos@imb-cnm.csic.es)*


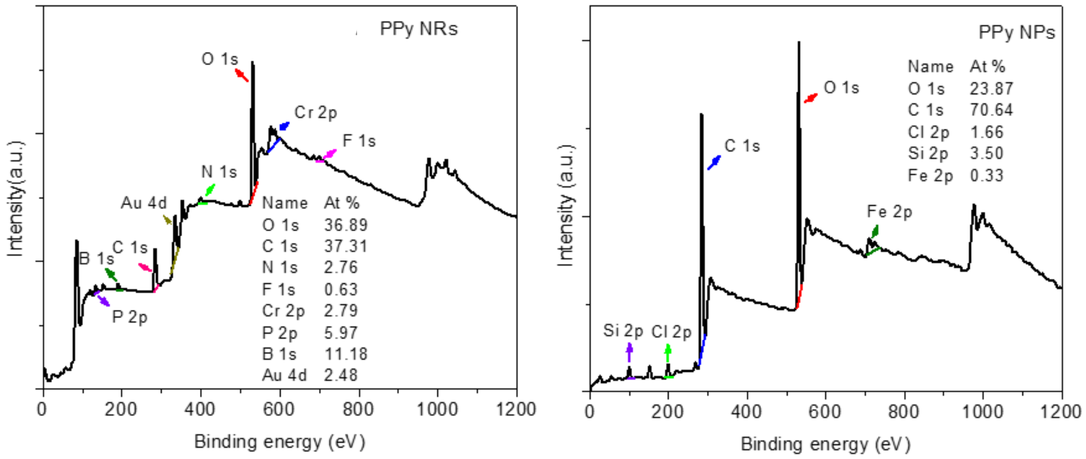


**Figure S1.** Survey XPS spectra for the PPy NRs and PPy NPs films and estimation of the concentration of these elements at the surface. The presence of F, Cr, P, B and Au in the electrochemically deposited PPy NRs is attributed to the reactants used in the synthesis process or etching of the template. Similarly the presence of Cl, Fe and Si is attributed to the reactants used in the chemical synthesis and the substrate (see experimental section).

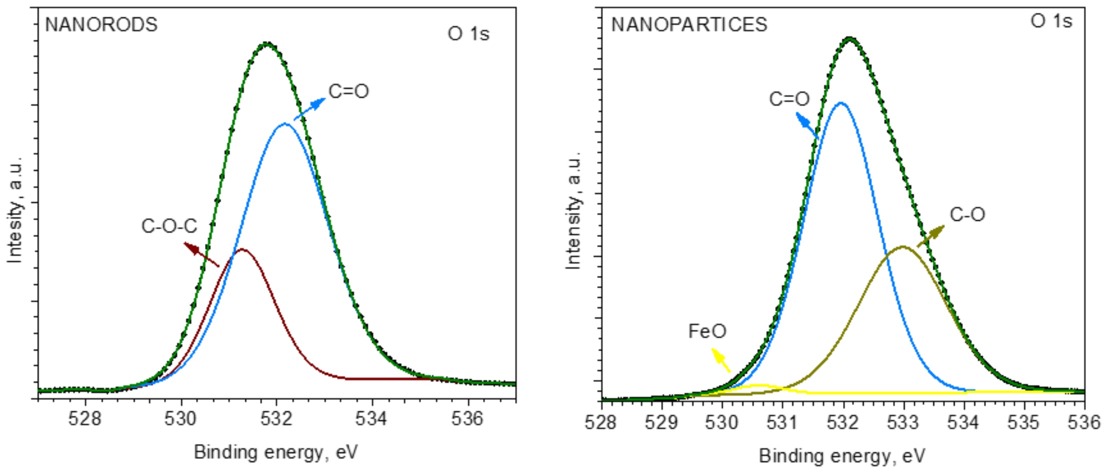


**Figure S2.** O 1s XPS core-level peaks recorded at the PPy NRs (left) and PPy NPs (right)

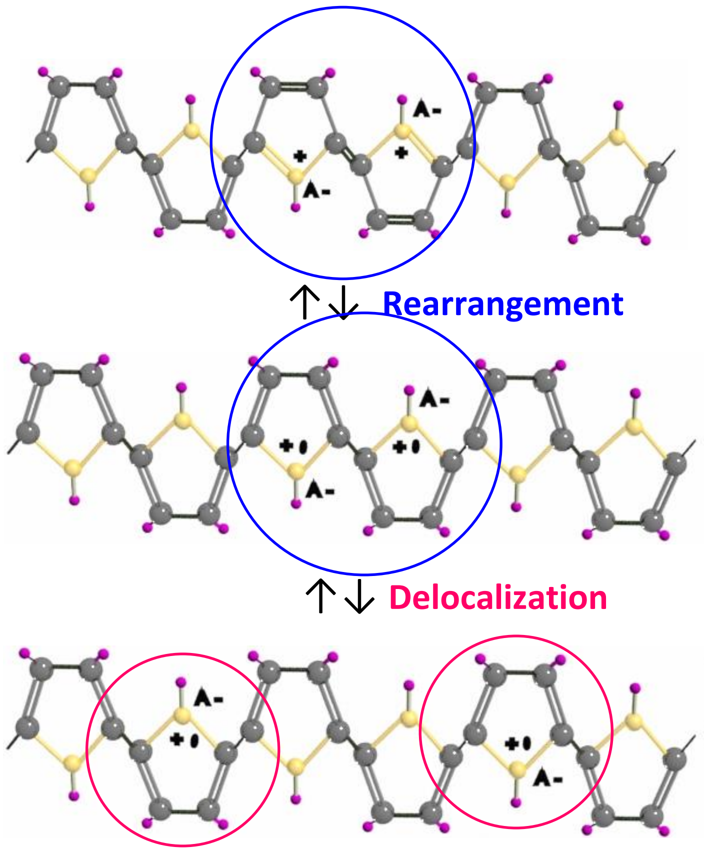


**Figure S3.** The transfer mechanism of bipolarons into polarons; the free holes of bipolarons are filled by the electron from donating analyte what is causing the transformation of bipolarons (two free holes) into polarons (one free hole, one electron); afterward, the rearrangement in PPy structure is occurred, what is followed with the bonds movement inside of Py ring and delocalization at the PPy chain.
